# Supplementary material for: LGBTQIA+ Adolescents’ Perceptions of Gender Tailoring and Portrayal in a Virtual-Reality-Based Alcohol-Prevention Tool: A Qualitative Interview Study and Thematic Analysis
Source: Int J Environ Res Public Health. 2023 Feb 4;20(4):2784. doi: 10.3390/ijerph20042784 (PMC9957229; doi:10.3390/ijerph20042784)
Supplement: Supplementary file 1 [file ijerph-20-02784-s001.zip › ijerph-2135211-supplementary.pdf]

**Supplementary Table S1**: Consolidated Criteria for Reporting Qualitative Studies (COREQ): 32-item checklist. (Table developed from: Tong, A.; Sainsbury, P.; Craig, J., Consolidated criteria for reporting qualitative research (COREQ): a 32-item checklist for interviews and focus groups. *Int J Qual Health Care* **2007**, 19, (6), 349-57) [39]).

| No | Item | Guide question/description | Content Reported | on page |
|---|---|---|---|---|
| **Domain 1: Research team and reflexibility** | | | | |
| Personal Characteristics | | | | |
| 1. | Interviewer/facilitator | Which author/s conducted the interview? | C.P. | 4 |
| 2. | Credentials | What were the researcher's credentials? e. g. PhD, MD | M.Sc. Public Health, M.A. Educational Science | 4 |
| 3. | Occupation | What was their occupation at the time of the study? | Research Associate | 4 |
| 4. | Gender | Was the researcher male or female? | female | 4 |
| 5. | Experience and Training | What experience or training did the researcher have? | experienced in conducting qualitative interviews through prior studies | 4 |
| Relationship with participants | | | | |
| 6. | Relationship established | Was a relationship established prior to study commencement? | None of the interviewees was prior known to the researcher. Participants responded to recruitment (presentation of the study in queer youth contexts and Instagram posts shared by youth organisations) themselves. Then a date, place, and time were arranged individually. | 5 |
| 7. | Participant knowledge of the interviewer | What did the participants know about the researcher? e. g. personal goals, reasons for doing the research | The researcher presented as an ally of the queer community within research and as the study's goal to further develop gender portrayal and tailoring within health promotion and prevention. Participants were aware of the purpose of the study though before out-handed study information sheets. | 5 |
| 8. | Interviewer characteristics | What characteristics were reported about the interviewer/facilitator? e. g. bias, assumptions, reasons, and interests in the research topic | The researcher presented a researcher and former social worker interested in further development of gender aspects within health promotion and prevention. | 5 |
| **Domain 2: study design** | | | | |
| Theoretical framework | | | | |
| 9. | Methodological orientation and Theory | What methodological orientation was stated to underpin the study? e. g. grounded theory, discourse analysis, ethnography, phenomenology, content analysis | A thematic analysis with an orientation towards reflexive thematic analysis by Braun and Clarke [37] with an inductive approach was applied. | 4 |
| Participant selection | | | | |

| | | | | |
|---|---|---|---|---|
| 10. | Sampling | How were participants selected? e. g. purposive, convenience, consecutive, snowball | Convenience sample | 4 |
| 11. | Method of approach | How were participants approached? e. g. face-to-face, telephone, mail, email | Potential participants were approached via the personal presentation of the study in queer youth contexts and via Instagram posts shared by queer youth organisations. | 4 |
| 12. | Sample size | How many participants were in the study? | 16 | 1, 7 |
| 13. | Non-participation | How many people refused to participate or dropped out? Reasons? | Two interested participants were aged older than 19 years and thus did not meet including criteria | - |
| Setting | | | | |
| 14. | Setting of data collection | Where was the data collected? e. g. home, clinic, workplace | Interviews were conducted at the Institute of Health and Nursing Science, Charité – Universitätsmedizin Berlin and upon wish by participants in collaborating youth clubs. | 4 |
| 15. | Presence of non-participants | Was anyone else present besides the participants and researchers? | No one else was present. | 4 |
| 16. | Description of sample | What are the important characteristics of the sample? e. g. demographic data, date | Inclusion criteria are presented in the methods section; participants´ characteristics are presented in the results section and Table 1. Interviews took place between January and August 2022. | 4, 7 |
| Data collection | | | | |
| 17. | Interview guide | Were questions, prompts, guides provided by the authors? Was it pilot tested? | The interview guide is described in the paper. It was pilot tested with colleagues and a similar version was used in a prior study [16]. | 5 |
| 18. | Repeat interviews | Were repeat interviews carried out? If yes, how many? | No repeated interviews. | - |
| 19. | Audio/visual recording | Did the research use audio or visual recording to collect the data? | Interviews were digitally audio-recorded. | 5 |
| 20. | Field notes | Were field notes made during and/or after the interview or focus group? | Field notes were made after the interviews, including observations and ideas. | 5 |
| 21. | Duration | What was the duration of the interviews or focus group? | Interviews´ duration ranged between 18 to 77 minutes with an average duration of 46 minutes. | 7 |
| 22. | Data saturation | Was data saturation discussed? | Data saturation is discussed within the methods and limitations section | 2, 15 |
| 23. | Transcripts returned | Were transcripts returned to participants for comment and/or correction? | Transcripts were not returned. | |
| **Domain 3: analysis and findings** | | | | |
| Data analysis | | | | |

| | | | | |
|---|---|---|---|---|
| 24. | Number of data coders | How many data coders coded the data? | C.P. and K.H. coded the data of the 3 interviews appearing as "the richest" to the interview conductor C.P. The remaining 13 interviews were coded by C.P. alone. | 6 |
| 25. | Description of the coding tree | Did authors provide a description of the coding tree? | The coding tree is presented as the final thematic map in the results and illustrated graphically in figure 1. | 7 |
| 26. | Derivation of themes | Were themes identified in advance or derived from the data? | Themes were identified inductively from the data. | 5 |
| 27. | Software | What software, if applicable, was used to manage the data? | MAXQDA software (2022) (VERBI Software GmbH, Berlin, Germany) was used for computer-assisted organisation and analysis of transcripts. | 5 |
| 28. | Participant checking | Did participants provide feedback on the findings? | The paper was sent to participants prior to publishing to enable providing feedback on the findings. One participant provided positive feedback. | 5 |
| Reporting | | | | |
| 29. | Quotations presented | Were participant quotations presented to illustrate the themes/findings? Was each quotation identified? e. g. participant number | Participant quotations are presented to illustrate the findings. Each quotation is identified by a participant letter (randomly attributed to all participants during pseudonymisation). | 8-13 |
| 30. | Data and findings consistent | Was there consistency between the data presented and the findings? | Results are supported by data with direct quotations of participants. | 8-13 |
| 31. | Clarity of major themes | Were major themes clearly presented in the findings? | Major themes are presented, explained, and illustrated by direct and indirect quotations of participants in the results, also graphically in figure 1. | 7-13 |
| 32. | Clarity of minor themes | Is there a description of diverse cases or discussion of minor themes? | Each code and sub-code is presented, named, explained, and illustrated by at least one direct quotation of participants in the results, also graphically in figure 1. Diverse cases and opinions are described throughout the results section. | 7-13 |
